# Induction of Cyp450 enzymes by 4-thiazolidinone-based derivatives in 3T3-L1 cells *in vitro*

**DOI:** 10.1007/s00210-020-02025-7

**Published:** 2020-11-21

**Authors:** Konrad A. Szychowski, Bartosz Skóra, Anna Kryshchyshyn-Dylevych, Danylo Kaminskyy, Kamila Rybczyńska-Tkaczyk, Roman Lesyk, Jan Gmiński

**Affiliations:** 1 Department of Lifestyle Disorders and Regenerative Medicine, University of Information Technology and Management in Rzeszow, Sucharskiego 2, 35-225 Rzeszow, Poland; 2 Department of Pharmaceutical, Organic and Bioorganic Chemistry, Danylo Halytsky Lviv National Medical University, Pekarska 69, Lviv, 79010 Ukraine; 3 Department of Environmental Microbiology, University of Life Sciences, Leszczyńskiego 7, 20-069 Lublin, Poland

**Keywords:** CYP450, 4-Thiazolidinone, Cyp1a1, 3T3-L1 cells, Cyp1b1, AhR

## Abstract

4-Thiazolidinones and related derivatives are regarded as privileged structures in medicinal chemistry and a source of new drug-like compounds. To date it is known that thiazolidinones are able to induce CYP1A1 activity in 3T3-L1 cells. Therefore, to extend the knowledge of the mechanism of thiazolidinones in the cell, four chemically synthesized heterocycles were tested on 3T3-L1 cells. The 3T3-L1 cells were exposed to Les-2194, Les-3640, Les-5935, and Les-6166. Our study showed that 1 μM βNF, Les-2194, and Les-6166 decreased the expression of *Ahr* mRNA. In turn, βNF, Les-2194, and Les-3640 increased the *Cyp1a1* mRNA expression at the same time interval. On the other hand, Les-5935 was found to decrease the *Cyp1a1* mRNA expression. Interestingly, the expression of *Cyp1a2* mRNA was activated only by βNF and Les-2194. The expression of *Cyp1b1* mRNA in the 3T3 cell line increased after the βNF and Les-2194 treatment but declined after the exposure to Les-5935 and Les-6166. Moreover, the Les-2194 and Les-5935 compounds were shown to increase the activity of EROD, MROD, and PROD. Les-3640 increased the activity of EROD and decreased the activity of PROD. In turn, the treatment with Les-6166 resulted in an increase in the activity of EROD and a decrease in the activity of MROD and PROD in the 3T3-L1 cells.

**Supplementary Information:**

The online version contains supplementary material available at 10.1007/s00210-020-02025-7.

## Introduction

4-Thiazolidinones and related derivatives are regarded as privileged structures in medicinal chemistry and are a source of new drug-like compounds (Tripathi et al. 2014; Kaminskyy et al. 2017a). In terms of modern medicinal chemistry, 4-thiazolidinones possess a variety of biological activities (both in screening campaigns and directed experiments). They are also regarded as a tool for synthesis of related heterocycles, simplified analogs, and diverse complex heterocycles in various approaches. Moreover, among the variety of thiazolidinone subtypes, 5-ene-4-thiazolidinones are of special interest as hit and lead compounds with anticancer, antimicrobial, antiviral, and antitrypanosomal activities (Kaminskyy et al. 2017a; Lesyk 2020a, b). However, a new trend in medicinal chemistry refers to these compounds as frequent hitters or pan-assay interference compounds, which are useless due to their possible low selectivity. This is challenged by the Michael acceptor property of 5-ene-4-thiazolidinones (Kaminskyy et al. 2017b). Such a thesis is actively discussed in the literature and requires further investigation. Nevertheless, we established earlier that annealing of the thiazolidine core into fused analogs (thiopyrano[2,3-*d*]thiazoles, chromeno[2,3-*d*]thiazoles, thiazolo[4,5-*b*]pyridine, etc.) is one of the prominent optimization directions that helps to conserve the activity pattern of synthetic precursors (5-ene-4-thiazolidinones), decrease the toxicity, and avoid the Michael acceptor properties as well (Kryshchyshyn et al. 2018).

To date, it has been described that 4-thiazolidinones derivates can be peroxisome proliferator-activated receptor (PPAR) agonists (Wright et al. 2014; Zhou et al. 2015; Szychowski et al. 2017a). PPARs are members of the nuclear receptor superfamily that can be activated by various natural fatty acids and xenobiotics (Berger et al. 2005; Scatena et al. 2008; Wojtowicz et al. 2014). Three subtypes of PPARs, i.e., PPARα, PPARβ, and PPARγ, have been identified (Lea et al. 2004; Scatena et al. 2008; Tachibana et al. 2008).

After activation, PPARs bind with the retinoid X receptor (RXR) and form PPARs/RXR heterodimers, which regulate transcription of genes involved in different metabolic pathways (Bardot et al. 1993). Similarly, the aryl hydrocarbon receptor (AhR) requires participation of RXR for activation of gene expression (Hessel-Pras et al. 2018). Moreover, Wójtowicz et al. (2017) described crosstalk between AhR and PPARγ receptors in mouse neurons. Similarly, as reported by Kim et al. (2008), troglitazone (PPARγ agonist) affects induction of Cyp1a1, whose expression is AhR dependent, in murine hepatoma Hepa-1c1c7 cells. It has also been described that alpha-naphthoflavone (αNF), which is an AhR antagonist, inhibits differentiation of 3T3-L1 pre-adipocytes, which is a well-documented PPARγ-dependent process (He et al. 2013). To date, PPARγ activators have been reported to act as carcinogens as well, as described in some papers (Scatena et al. 2008).

It is well known that many xenobiotics can accumulate in adipose tissue (Barouki 2013). Unfortunately, the consequences of this accumulation are still poorly understood (Jackson et al. 2017). Moreover, adipose tissue can also metabolize xenobiotics to a small extent with the involvement of cytochrome P450 enzymes (Ellero et al. 2010), e.g., CYP1A1, which has been examined extensively for its capacity to activate compounds with carcinogenic properties (Androutsopoulos et al. 2009). Exposure to chemicals and environmental carcinogens is thought to increase the level of CYP1A1 expression in extrahepatic tissues through the AhR receptor (Nebert et al. 2000; Di Bello et al. 2007). Dysregulation of these pathways may be implicated in tumor progression (Androutsopoulos et al. 2009). Similarly CYP1B1, which is expressed in liver and extrahepatic tissues, carries out the metabolism of numerous xenobiotics, including the metabolic activation of polycyclic aromatic hydrocarbons (Spink et al. 1998). Moreover, CYP1B1 has also been shown to be important in regulating endogenous metabolic pathways, including the metabolism of steroid hormones, fatty acids, melatonin, and vitamins (Li et al. 2017). CYP1B1 and its interaction with PPARs, estrogen receptors (ERs), and retinoic acid receptors (RARs) contribute to the maintenance of the homeostasis of these endogenous compounds (Angus et al. 1999; Nebert and Dalton 2006). Accumulating data indicate that modulation of CYP1B1 can decrease adipogenesis and tumorigenesis and prevent obesity, hypertension, atherosclerosis, and cancer (Malik et al. 2012; Song et al. 2016; Li et al. 2017). Therefore, it may be feasible to consider CYP1B1 as a therapeutic target for the treatment of metabolic diseases (Li et al. 2017; Alzahrani and Rajendran 2020).

The molecular mode of the anticancer action of the abovementioned compounds is still unclear, but it is believed to be related with PPAR pathways (Lu et al. 2011; Asati et al. 2014). Our previous study has demonstrated that 4-thiazolidinone-based derivatives (thiopyrano[2,3-*d*]thiazole derivative Les-2194 and thiazolidinone-pyrazoline hybrid molecule Les-3640) cause apoptotic cell death in human squamous carcinoma cells (SCC-15) with key involvement of PPARγ (Szychowski et al. 2017a, b). Moreover, another compound studied by our team 5*Z*-(4-fluorobenzylidene)-2-(4-hydroxyphenylamino)-thiazol-4-one (Les-236) has been found to exhibit broad cytotoxic and proapoptotic action in SCC-15, lung carcinoma (A549), colon adenocarcinoma (CACO-2), neuroblastoma (SH-SY5Y), and skin fibroblast (BJ) cell lines (Szychowski et al. 2019).

At present, there are very limited data on the influence of anticancer 4-thiazolidinone-based derivatives on Cyp1a1, Cyp1b1, Cyp1a2, and Cyp2b10 expression or activity. Moreover, the impact of Les-2194, Les-3640, Les-5935 (chromeno[2,3-*d*]thiazole derivative), or Les-6166 (thiazolidinone-indole hybrid) on induction of Cyp1a1, Cyp1b1, Cyp1a2, and Cyp2b10 expression or activity has never been studied. These compounds represent the most promising classes of 4-thiazolidinone-based derivatives as potential anticancer agents. In particular, Les-2194 (Havrylyuk et al. 2012), Les-3640 (Kryshchyshyn et al. 2012), and Les-5935 (Table S1, ESI) inhibited the growth of sixty cancer cell lines representing nine neoplastic diseases at sub-micromolar and micromolar concentrations according to US NCI protocols (Shoemaker 2006).

Due to the lipophilic nature of the studied compounds, the mouse non-cancerous preadipocyte 3T3-L1 cell line was selected for metabolism studies. In addition, it is possible that they accumulate in adipose tissue. Therefore, the aim of our study was to determine whether the 4-thiazolidinone-based derivatives induce the expression and/or activity of Cyp1a1, Cyp1b1, Cyp1a2, and Cyp2b10 in the 3T3-L1 cell line.

## Materials and methods

### Reagents

Dulbecco’s Modified Eagle’s Medium (DMEM) without phenol red (10-013CV) and phosphate-buffered saline without calcium and magnesium (PBS) (21-040-CVR) were purchased from Corning (Manassas, VA, USA). Trypsin, penicillin, streptomycin, amphotericin B, β-naphthoflavone (βNF) (N3633), and dimethyl sulfoxide (DMSO) were purchased from Sigma-Aldrich (St. Louis, MO, USA). Substrates for P450 cytochrome ethoxyresorufin-O-deethylase (EROD) (5725-91-7), 7-methoxyresorufin O-demethylation (MROD) (5725-89-3), and pentoxyresorufm O-dealkylase (PROD) (87687-03-04) were supplied by Tocris Bioscience. Fetal bovine serum (FBS) (E5050-02), RNA isolation kits (E0309), and fast probe qPCR master mix (Rox) (E0423) were purchased from EURx (Gdańsk, Poland). TaqMan probes corresponding to specific genes encoding *Gapdh* (Mm99999915_g1), *Ahr* (Mm01291777_m1), *Cyp1a1* (Mm00487218_m1), *Cyp1b1* (Mm00487229_m1), *Cyp1a2* (Mm00487224_m1), and *Cyp2b10* (Mm01972453_s1) were purchased from Thermo Fisher Scientific (Grand Island, NY, USA).

Synthesis and physicochemical data of the tested compounds were described previously: Les-2194, *rel*-*N*-(2,4-dichlorophenyl)-2-[(5aR,11bR)-2-oxo-5a,11b-dihydro-2*H*,5*H*-chromeno[4',3':4,5]thiopyrano[2,3-*d*][1,3]thiazol-3(6*H*)-yl]acetamide (Kryshchyshyn et al. 2012), and Les-3640, 3-{2-[5-(4-dimethylaminophenyl)-3-phenyl-4,5-dihydropyrazol-1-yl]-4-oxo-4,5-dihydro-1,3-thiazol-5-ylidene}-2,3-dihydro-1*H*-indol-2-one (Havrylyuk et al. 2012). Spectral and analytical data for Les-5935 (9*H*-benzo[5,6]chromeno[2,3-*d*][1,3]thiazol-9-one) and Les-6166 (methyl 5-fluoro-3-[2-(4-hydroxyanilino)-4-oxo-4,5-dihydro-1,3-thiazol-5-ylidenmethyl]-1*H*-2-indolecarboxylate) are described in the Supplementary information.

All stock solutions were prepared by dissolving the compounds in DMSO. The final concentration of DMSO in the culture medium was always 0.1%.

### Synthesis of anticancer 4-thiazolidinone-based derivatives

The synthesis of Les-2194 and Les-3166 was performed according to the method described by Kryshchyshyn et al. (2012) and Havrylyuk et al. (2012), respectively.

#### General procedure for the synthesis of 9H-benzo[5,6]chromeno[2,3-d][1,3]thiazol-9-one (Les-5935)

Mixtures of 4-aminothiazol-2(5*H*)-one (5 mmol), 2-hydroxynaphthalene-1-carbaldehyde (5 mmol), and sodium acetate (5 mmol) were heated at reflux for 2 h in a mixture of AcOH and Ac_2_O (10 mL, 1:1). After cooling, the precipitate formed was filtered off, washed with AcOH, H_2_O, and EtOH, and recrystallized from a mixture of DMF/EtOH (2:1). Spectral and analytical data for compound Les-5935 is provided in supplementary material 1.

#### General procedure for the synthesis of methyl 5-fluoro-3-[2-(4-hydroxyanilino)-4-oxo-4,5-dihydro-1,3-thiazol-5-ylidenmethyl]-1H-2-indolecarboxylate (Les-6166)

A mixture of 0.1 mole of 4-hydroxyphenylthiourea (0.1 mol), methyl 5-fluoro-3-formylindole-2-carboxylate (0.11 mol), chloroacetic acid (0.1 mol), and fused sodium acetate (0.1 mol) in 100 mL of acetic acid was heated under reflux for 3 h. The crystalline precipitate was filtered off, washed with acetic acid, water, ethanol, and diethyl ether, and then recrystallized from a mixture DMF-acetic acid (1:2). Spectral and analytical data for compound Les-6166 is provided in supplementary material 1.

### 3T3-L1 cell culture

Mouse embryonic fibroblast cell line 3T3-L1 was obtained from the American Type Culture Collection (ATCC, distributor: LGC Standards, Łomianki, Poland). The 3T3-L1 cell line was maintained in phenol red-free DMEM supplemented with 10% FBS, 100 U/mL penicillin, 0.10 mg/mL streptomycin, and 250 ng/mL amphotericin B. The cells were maintained at 37 °C in a humidified atmosphere with 5% CO_2_. They were seeded in 96-well culture plates at a density of 6 × 10^3^ (for the 24-h treatment), 5 × 10^3^ (for the 48-h treatment), and 4 × 10^3^ (for the 72-h treatment) per well and initially cultured before the experiment for 24 h. Subsequently, the medium was replaced with a fresh one by raising the concentrations (100 nM, 1 μM, 10 μM, 50 μM, and 100 μM) of Les-2194, Les-3640, Les-5935, and Les-6166 (resazurin reduction and caspase-3 activity).

### Resazurin reduction assay

The resazurin reduction assay was used to determine the rate of cell metabolism and, based on this, cell viability (Rampersad 2012). Resazurin is a redox dye showing colorimetric and fluorometric changes depending on the cell metabolic activity. The resazurin reduction assay is based on the ability of metabolically active cells to convert blue resazurin (non-fluorescent form) to red resorufin (fluorescent form). To determine viability, the cells were seeded in 96-well plates. They were incubated in different series of the Les-2194, Les-3640, Les-5935, and Les-6166 compounds (100 nM, 1 μM, 10 μM, 50 μM, and 100 μM) for 24 h, 48 h, and 72 h at 37 °C. Next, the dilutions of the compounds were removed, and the DMEM medium containing 1% FBS and 10% of resazurin was added. Then, after 30 and 60 min of incubation, fluorescence measurements were made using a microplate reader (FilterMax F5). The maximum excitation spectrum was 530 nm, and the emission was 590 nm. The data were analyzed using Multi-Mode Analysis software and normalized to the fluorescence in the vehicle-treated control (% of control).

### Caspase-3 activity

Caspase-3 activity was used as a marker of cell apoptosis and was assessed according to Nicholson et al. (1995). To measure caspase-3 activity, the cells were seeded on 96-well plates and exposed to Les-2194, Les-3640, Les-5935, and Les-6166. After thawing (− 80 °C), the cells were lysed using lysis buffer (50 mM HEPES, pH 7.4, 100 mM NaCl, 0.1% CHAPS, 1 mM EDTA, 10% glycerol, and 10 mM DTT) at 10 °C for 10 min. The lysates were incubated in the caspase-3 substrate Ac-DEVD-pNA at 37 °C. Cells treated with 1 μM staurosporine were used as a positive control (data not shown). After 30 min, the absorbance of the lysates at 405 nm was measured using a microplate reader (FilterMax F5). The amount of the colorimetric product was continuously monitored for 120 min. The data were analyzed using Multi-Mode Analysis software and normalized to the absorbance in the vehicle-treated cells.

### qPCR analysis of mRNAs specific to genes encoding *Ahr*, *Cyp1a1*, *Cyp1b1*, *Cyp1a2*, and *Cyp2b10*

The quantitative polymerase chain reaction (qPCR) method was chosen to determine whether the tested compounds increase gene expression. The experiment was conducted with a procedure described previously (Szychowski et al. 2017a). For the qPCR assay, 3T3-L1 cells were seeded onto 12-well plates and initially cultured for 24 h. After 6-h exposure to 1 μM βNF, Les-2194, Les-3640, Les-5935, or Les-6166, the samples were collected, and total RNA was extracted from the 3T3-L1 cells using a RNA isolation kit (EURx, Gdańsk, Polska) according to the manufacturer’s instructions. Both the quality and quantity of the RNA were determined spectrophotometrically at 260 and 280 nm, respectively (ND/1000 UV/Vis; Thermo Fisher NanoDrop, USA). Two-step real-time reverse transcription (RT)-PCR was conducted with both the RT reaction and the quantitative PCR (qPCR) run using the CFX real-time system (Bio-Rad, USA). The RT reaction was carried out at a final volume of 20 μL with 800 ng RNA (as a cDNA template) using the cDNA reverse transcription kit in accordance with the manufacturer’s instructions. Products of the RT reaction were amplified using the fast probe qPCR master mix (EURx) with TaqMan probes as primers for specific genes encoding *Gapdh*, *Ahr*, *Cyp1a1*, *Cyp1b1*, *Cyp1a2*, and *Cyp2b10*. The amplification was carried out in a total volume of 20 μL containing 1× fast probe qPCR master mix (EURs) and 1.0 μL of the RT product, which was used as the PCR template. The standard qPCR procedures were carried out as follows: 2 min at 50 °C and 10 min at 95 °C, followed by 45 cycles of 15 s at 95 °C and 1 min at 60 °C. The threshold value (Ct) for each sample was set during the exponential phase, and the ΔΔCt method was used for data analysis. *Gapdh* was used as the reference gene.

### CYP450 activities (EROD, MROD, and PROD assays)

To confirm the results of the qPCR reaction and their translation into functional protein, the activity of the studied cytochromes was tested. We estimated the activity of the Cyp1a1/Cyp1b1, Cyp1a2, and Cyp2b1 enzymes using the fluorometric ethoxyresorufin-O-deethylase (EROD), 7-methoxyresorufin O-demethylation (MROD), and pentoxyresorufm O-dealkylase (PROD) substrates, respectively. The fluorescence EROD, MROD, and PROD assays were performed according to the method proposed by Kennedy et al. (1993). For the assays, the cells were seeded on 12-well plates and initially cultured for 24 h. Measurement of the EROD, MROD, and PROD activity was performed after 24 or 48 h of exposure to 1 μM βNF, Les-2194, Les-3640, Les-5935, and Les-6166. To perform the EROD, MROD, and PROD assays, lysed cells were transferred into multiwell plates, and the fluorescent product resorufin was quantified within the wells with a fluorescence plate reader (FilterMax F5) at an excitation wavelength of 530 nm and an emission wavelength of 590 nm. The protein concentration was determined spectrophotometrically in triplicate for each sample at 280 nm using the ND/1000 UV/Vis Thermo Fisher NanoDrop device.

### Statistical analysis

The data are presented as means ± SD of three independent experiments. Each treatment was repeated six times in each independent experiment (*n* = 18). The data were analyzed with one-way analysis of variance (ANOVA) followed by Tukey’s multiple comparison procedure ***P < 0.001, **P < 0.01, and *P < 0.05 vs. the control.

## Results

### Synthesis of 4-thiazolidinone-based derivatives

**Fig. 1:**
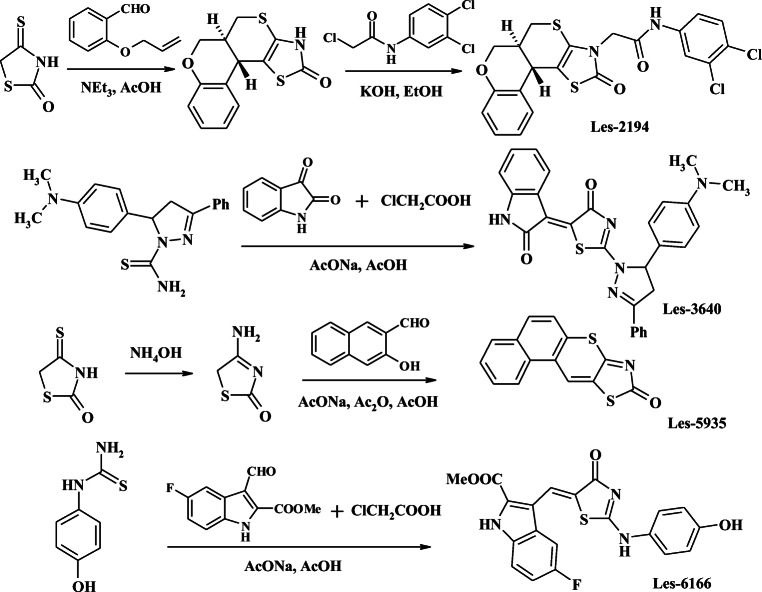
Synthesis of target thiazolidinone-based compounds. Les-2194 is described in Kryshchyshyn et al. (2012), and Les-3640 is described in Havrylyuk et al. (2012)

The general methods for the synthesis of the target thiazolidinone-based compounds Les-2194, Les-3640, Les-5935, and Les-6166 are presented in Fig. 1. Les-2194 was obtained *via N*-alkylation of *rel*-(5a*R*,11b*R*)-3,5a,6,11b-tetrahydro-2*H*,5*H*-chromeno[4',3':4,5]thiopyrano[2,3-d]thiazol-2-one by 2-chloro-*N*-(3,4-dichlorophenyl)-acetamide. Les-3640 and Les-6166 were synthesized in a one-pot three-component reaction involving [2+3]-cyclocondensation of appropriate *S*,*N*-binucleophile and chloroacetic acid followed by the Knoevenagel reaction with an appropriate oxo compound. In the case of Les-3640, 3-phenyl-5-(dimethylaminophenyl)-1-thiocarbamoyl-2-pyrazoline was used as the *S*,*N*-binucleophile, and isatin was used as the oxo compound; for the Les-6166 synthesis, 4-hydroxyphenylthiourea and methyl 5-fluoro-3-formylindole-2-carboxylate were used, respectively. The chromeno[2,3-*d*]thiazole Les-5935 was obtained *via* one-pot condensation of 4-aminothiazol-2(5H)-one and 2-hydroxynaphthalene-1-carbaldehyde.

### Measurement of resazurin reduction

Several thiazolidinone-based compounds have been previously shown to impact the viability of tumor cells (Szychowski et al. 2017a,b, 2019). Resazurin reduction experiments were performed to measure the effects of Les-2194, Les-3640, Les-5935, and Les-6166 on the viability of 3T3-L1 preadipocytes.

**Fig. 2:**
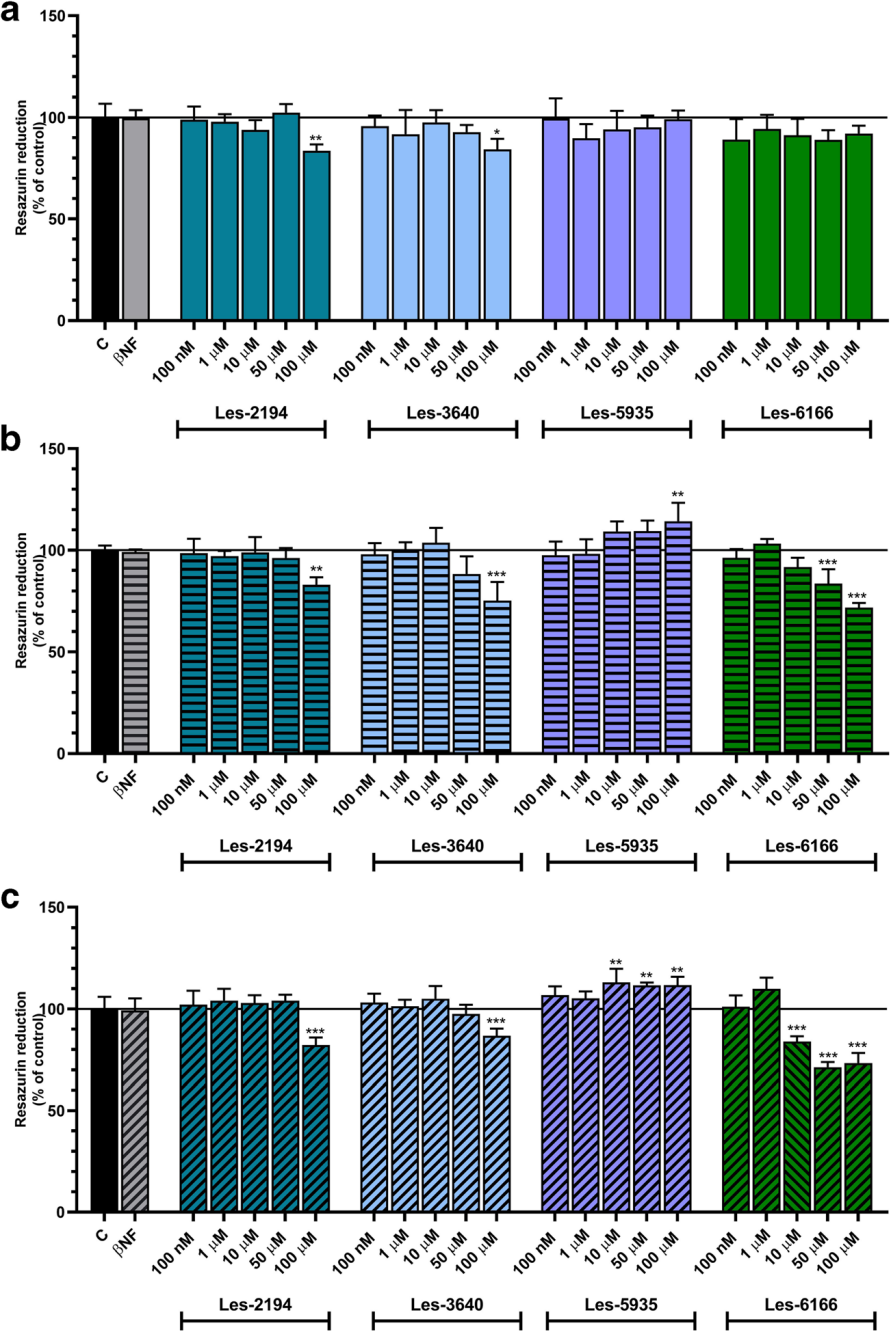
Effect of 100 nM, 1 μM, 10 μM, 50 μM, or 100 μM of Les-2194, Les-3640, Les-5935, or Les-6166 on resazurin reduction after 24 h (**a**), 48 h (**b**), and 72 h (**c**) of exposure of 3T3-L1 fibroblasts. Control vehicle only and 1 μM β-naphthoflavone (βNF) were included in the experiments. Data are expressed as means ± SD of three independent experiments, each of which consisted of six replicates per treatment group

After the 24-h exposure to 100 nM, 1 μM, 10 μM, 50 μM, and 100 μM of Les-2194, Les-3640, Les-5935, or Les-6166, only 100 μM of Les-2194 and Les-3640 induced a decrease in resazurin reduction in the 3T3-L1 cells (a decrease by 16.43 and 15.77%, respectively, compared to the control) (Fig. 2a).

Similarly, after the 48-h exposure to 100 nM, 1 μM, 10 μM, 50 μM, and 100 μM of Les-2194, Les-3640, Les-5935, or Les-6166, 100 μM of Les-2194 and Les-3640 caused a decrease in resazurin reduction in the 3T3-L1 cells (a decrease by 17.07 and 24.83% respectively, compared to the control). Moreover, Les-6166 decreased resazurin reduction at the concentrations of 50 μM and 100 μM (a decrease by 16.44 and 28.19%, respectively, compared to the control). In turn, compound Les-5935 at the concentration of 100 μM increased resazurin reduction by 14.19%, compared to the control (Fig. 2b).

After the 72-h exposure to the studied compounds, or Les-6166, similar as in the previous variant, 100 μM of Les-2194 and Les-3640 contributed to a decrease in resazurin reduction in the 3T3-L1 cells (a decrease by 17.88 and 13.20%, respectively, compared to the control). Les-6166 decreased resazurin reduction at the concentrations of 10 μM, 50 μM, and 100 μM (a decrease by 16.15, 28.77, and 26.65%, respectively, compared to the control). In turn, compound Les-5935 at the concentration of 10 μM, 50 μM, and 100 μM increased resazurin reduction by 13.12, 11.68, and 11.75%, respectively, compared to the control (Fig. 2c).

### Measurement of caspase-3 activity

To date, thiazolidinone-based compounds have been shown to initiate the apoptotic process in various cancerous cell lines (Szychowski et al. 2017a,b, 2019). Caspase-3 activity assay was performed to measure the effects of Les-2194, Les-3640, Les-5935, and Les-6166 on the apoptotic process in the 3T3-L1 cell line.

**Fig. 3:**
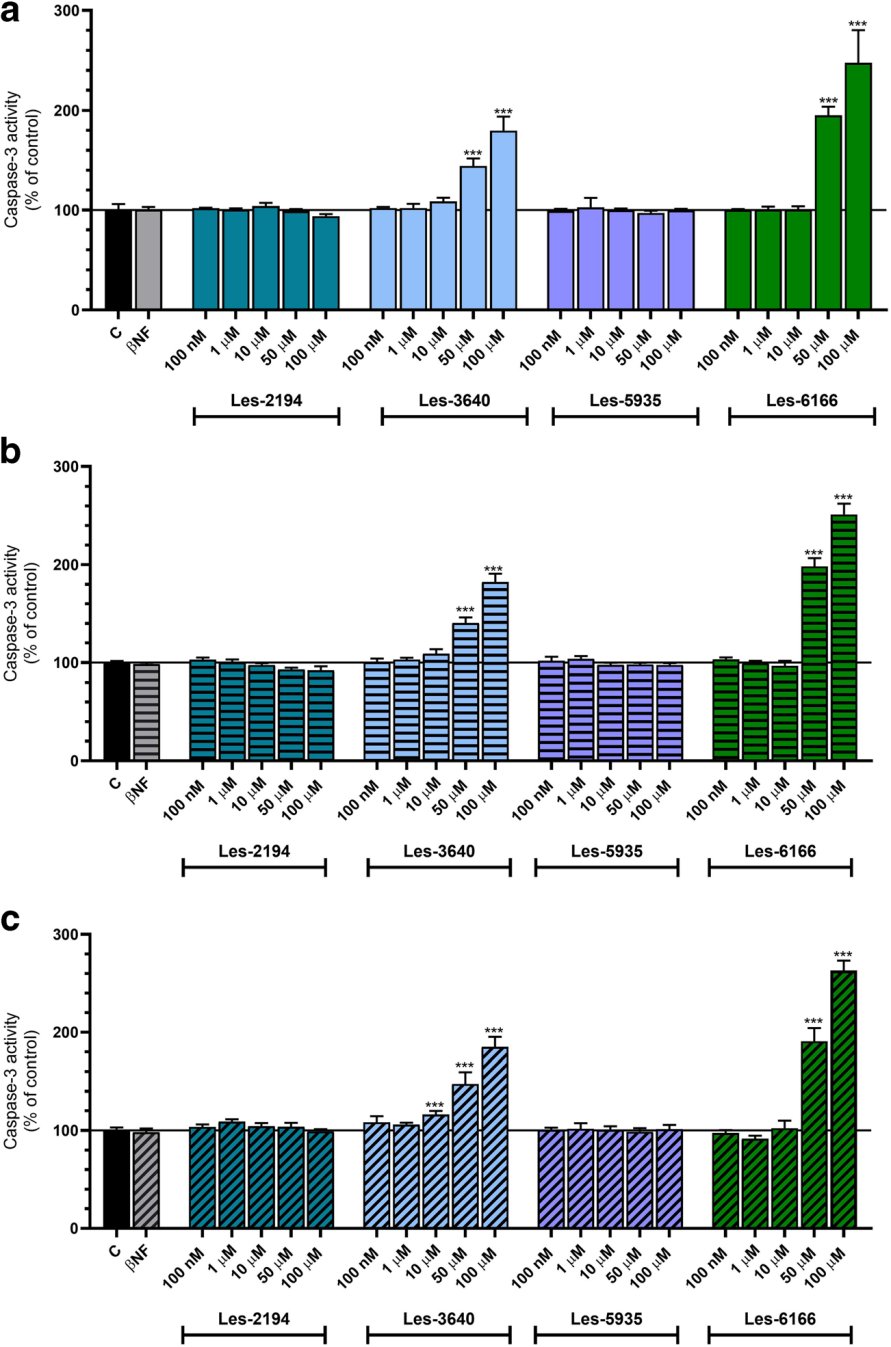
Effect of 100 nM, 1 μM, 10 μM, 50 μM, or 100 μM of Les-2194, Les-3640, Les-5935, or Les-6166 on caspase-3 activity after 24 h (**a**), 48 h (**b**), and 72 h (**c**) of exposure of 3T3-L1 fibroblasts (**a**). Control vehicle only and 1 μM β-naphthoflavone (βNF) were included in the experiments. Data are expressed as means ± SD of three independent experiments, each of which consisted of six replicates per treatment group

After the 24-h exposure to 100 nM, 1 μM, 10 μM, 50 μM, and 100 μM of Les-2194, Les-3640, Les-5935, or Les-6166, compounds Les-2194 and Les-5935 did not affect caspase-3 activity in the 3T3-L1 cells. Compounds Les-3640 and Les-6166 at the concentrations of 50 μM and 100 μM caused an increase in caspase-3 activity (an increase by 44.27 and 79.75%, respectively, for Les-3640 and an increase by 95.09 and 147.44%, respectively, for Les-6166) (Fig. 3a).

After the 48-h exposure to the studied compounds, as in the case of the 24-h exposure, compounds Les-2194 and Les-5935 did not affect caspase-3 activity in the 3T3-L1 cells. Moreover, compounds Les-3640 and Les-6166 at the concentrations of 50 μM and 100 μM induced an increase in caspase-3 activity (an increase by 40.33 and 82.33%, respectively, for Les-3640 and an increase by 98.26 and 151.47%, respectively, for Les-6166) (Fig. 3b).

Similarly, after the 72-h exposure to the studied compounds, Les-2194 and Les-5935 did not affect caspase-3 activity in the 3T3-L1 cells. Compound Les-6166 at the concentrations of 50 μM and 100 μM increased caspase-3 activity (an increase by 90.85 and 163.21%, respectively, compared to the control). Compound Les-3640 caused an increase in caspase-3 activity at the concentrations of 10 μM, 50 μM, and 100 μM (an increase by 16.18; 47.36 and 85.06%, respectively, compared to the control) (Fig. 3c).

### Expression of *Ahr*, *Cyp1a1*, *Cyp1b1*, *Cyp1a2*, and *Cyp2b10* mRNA

AhR and AhR-related genes are involved in the metabolism and detoxification system of different ligands, such as drugs, tetrachlorodibenzo-p-dioxin (TCDD), or βNF (Tian et al. 2015). To date, the Les-2194 and Les-3640 compounds have been shown to exhibit properties of PPARγ receptor agonists (Szychowski et al. 2017a, b). Therefore, due to crosstalk between AhR and PPARγ receptors, investigations of the mRNA expression of *Ahr* and *Ahr-*related genes were performed.

**Fig. 4:**
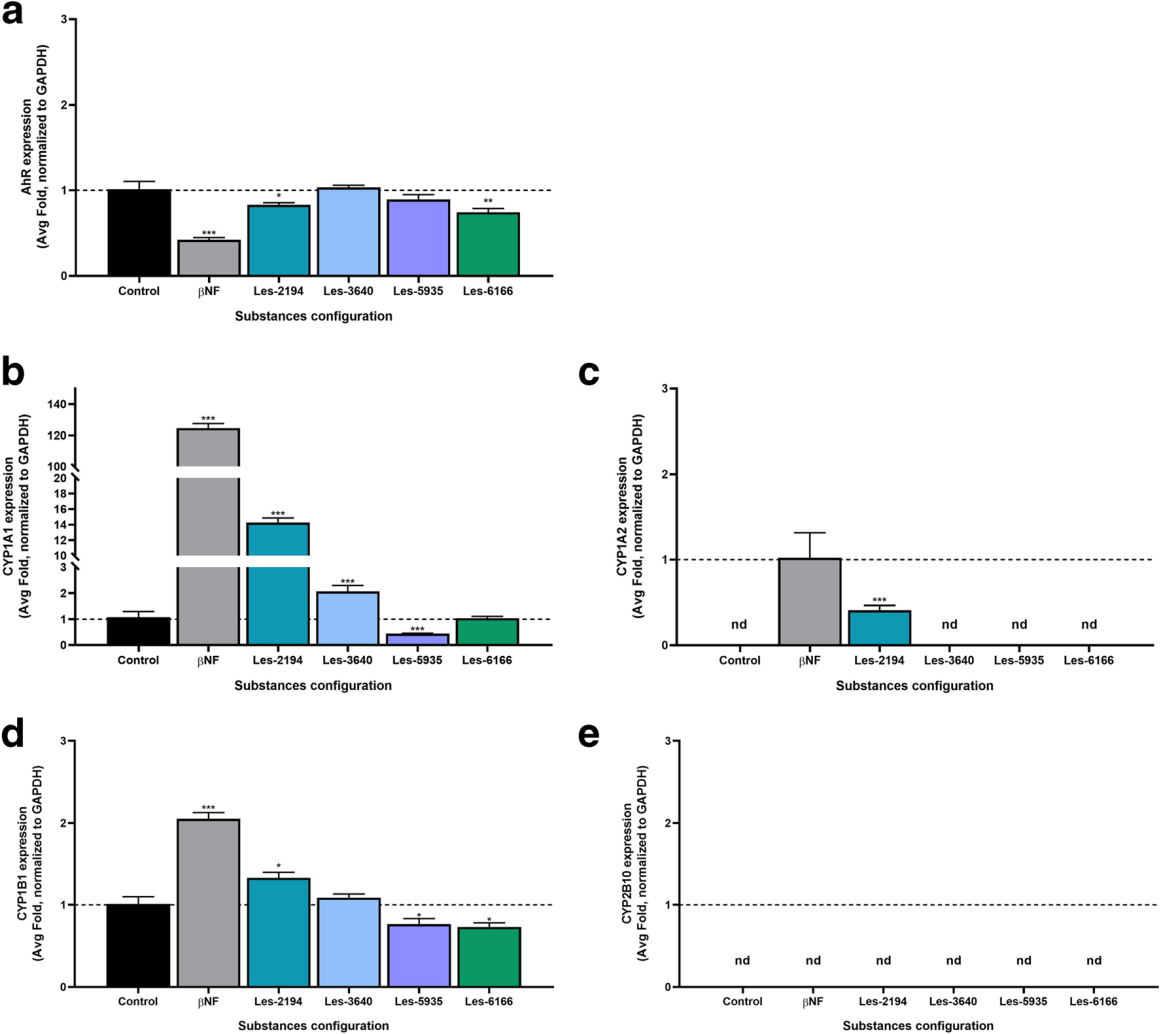
Effect of 1 μM Les-2194, Les-3640, Les-5935, or Les-6166 and 1 μM β-naphthoflavone (βNF) on the mRNA expression of *Ahr* (**a**), *Cyp1a1* (**b**), *Cyp1a2* (**c**), *Cyp1b1* (**d**), and *Cyp2b10* (**e**) genes after 6 h of exposure of 3T3-L1 fibroblasts. The lack of expression of *Cyp2b10* mRNA was confirmed by the use of mouse astrocyte cells as a positive control; see supplementary material 2 for details. Data are expressed as means ± SD of three independent experiments, each of which consisted of six replicates per treatment group. **P < 0.01 and ***P < 0.001 versus the control cells

The expression of *Ahr*, *Cyp1a1*, *Cyp1b1*, *Cyp1a2*, and *Cyp2b10* mRNA was measured after 6 h of exposure to βNF, Les-2194, Les-3640, Les-5935, or Les-6166. βNF, Les-2194, and Les-6166 were found to decrease the expression of *Ahr* mRNA by 57.97, 17.00, and 25.69% respectively, compared to the control (Fig. 4a). At the same time interval, βNF, Les-2194, and Les-3640 increased the *Cyp1a1* mRNA expression by 1240051.71, 1325.58, and 106.18%, respectively, compared to the control. In turn, Les-5935 decreased the *Cyp1a1* mRNA expression by 56.16%, compared to the control (Fig. 4b). Interestingly, the expression of *Cyp1a2* mRNA was activated only by βNF and Les-2194 (41.22% compared to the expression induced by βNF); therefore, βNF was regarded to be the control in this analysis (Fig. 4c). After the βNF and Les-2194 treatment, the expression of *Cyp1b1* mRNA in the 3T3 cell line increased by 105.11 and 33.04%, respectively, compared to the control. In turn, Les-5935 and Les-6166 decreased the expression of *Cyp1b1* mRNA by 23.66 and 27.02%, respectively, compared to the control (Fig. 4d). No expression of *Cyp2b10* mRNA was detected in the 3T3 cell line (Fig. 4e).

### CYP450 activities

Currently, it is well known that mRNA expression does not fully translate into its functions (Perl et al. 2017). Therefore, enzymatic activity assays EROD, MROD, and PROD were carried out to confirm or deny that changes in mRNA expression affect protein activity.

#### EROD assay

**Fig. 5:**
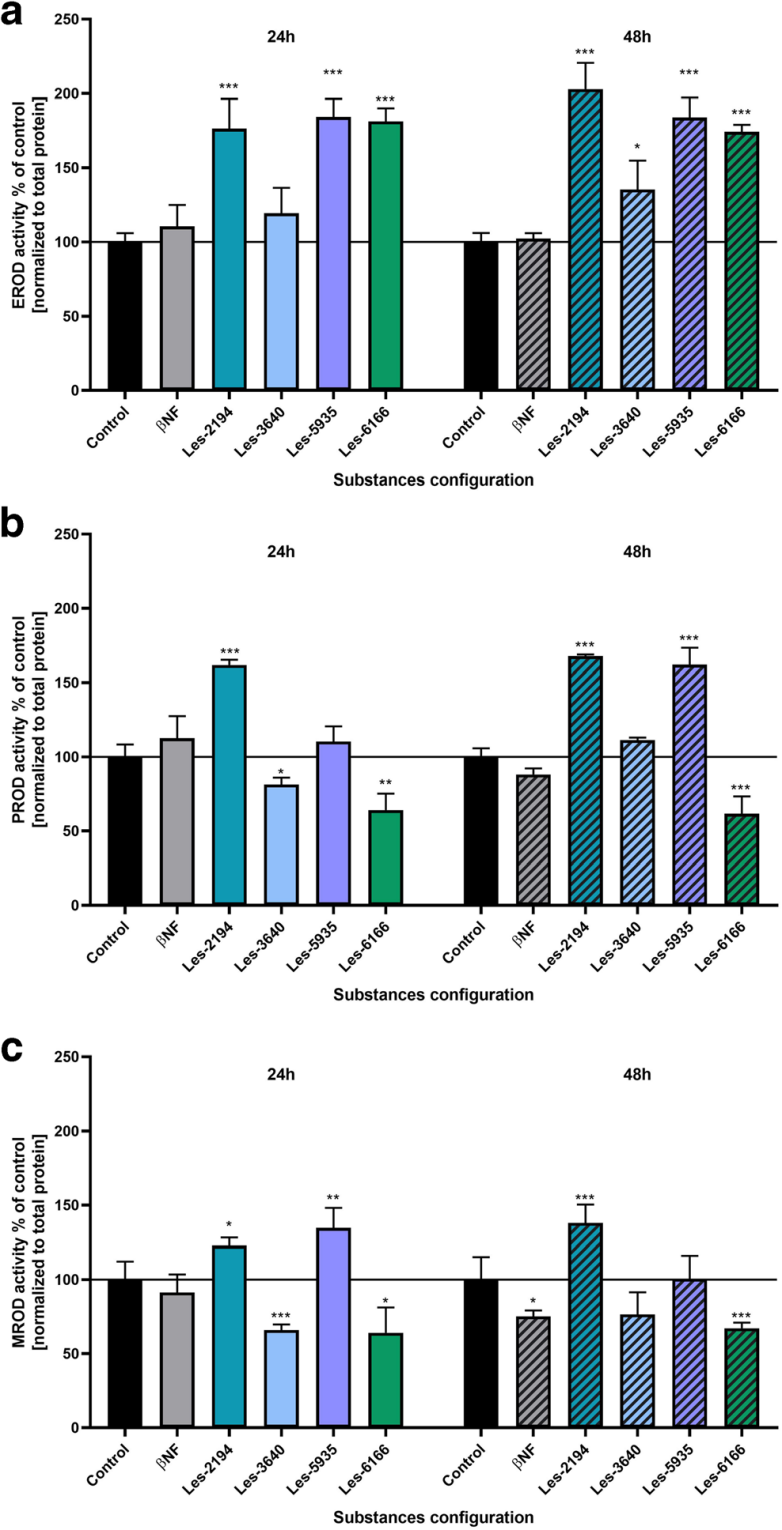
Effect of 1 μM Les-2194, Les-3640, Les-5935, or Les-6166 and 1 μM β-naphthoflavone (βNF) on EROD (**a**), MROD (**b**), and PROD (**c**) activities after 24 and 48 h of exposure of 3T3-L1 fibroblasts. Data are expressed as means ± SD of three independent experiments, each of which consisted of six replicates per treatment group. **P < 0.01 and ***P < 0.001 versus the control cells

After 24 and 48 h of exposure of the 3T3-L1 cells to 1 μM Les-2194, Les-5935, or Les-6166, we observed an increase in EROD activity. An increase by 76.45 and 102.90% compared to the control was observed in the case of Les-2194. Les-5935 increased the activity by 84.31 and 83.90%, compared to the control, whereas an 81.29 and 74.30% increase was induced by Les-6166, compared to control. In turn, Les-3640 increased the EROD activity only after 48 h (by 35.40% compared to the control) (Fig. 5a).

#### PROD assay

After 24 and 48 h of exposure of the 3T3-L1 cells to 1 μM Les-2194, the PROD activity was increased by 61.70 and 67.67%, respectively, compared to the control. After 24 h of exposure to Les-3640, we observed an 18.70% decrease in the PROD activity, compared to the control. After 48 h, Les-5935 increased the PROD activity by 61.95%, compared to the control. Les-6266 induced a decrease in the PROD activity at both time intervals (a decrease by 36.00 and 38.43%, compared to the control) (Fig. 5b).

#### MROD assay

βNF reduced the MROD activity only after 48 h of exposure (a decrease by 24.89%, compared to the control). Likewise previously, Les-2194 increased the MROD activity in the 3T3-L1 cells after 24 and 48 h (an increase by 22.76 and 37.98%, respectively, compared to the control). After 24-h exposure, Les-5935 increased the MROD activity by 34.64%, compared to the control. In turn, Les-3640 and Les-6166 contributed to a decrease in the MROD activity at both time intervals, i.e., by 34.10 and 23.55% in the case of Les-3640 and by 36.02 and 32.83% in the case of Les-6166, compared to the control (Fig. 5c).

## Discussion

The present experiments showed that, after 24, 48, and 72 h of exposure to 100 nM, 1 μM, 10 μM, 50 μM, and 100 μM, the Les-2194, Les-3640, and Les-6166 compounds caused a significant decrease in the resazurin reduction assay only at the highest micromolar concentrations in the 3T3-L1 cells. Interestingly, compound Les-5935 after 72 h of exposure in the concentration range from 10 to 100 μM induced an increase in the resazurin reduction assay. Moreover, at all the time intervals studied, compounds Les-2194 and Les-5935 did not affect caspase-3 activity in the 3T3-L1 cells. However, compounds Les-3640 and Les-6166 at the concentrations of 50 μM and 100 μM contributed to an increase in caspase-3 activity. Therefore, it can be assumed that the compounds applied in the range 100 nM to 1 μM neither are toxic nor initiate apoptosis in the normal mouse fibroblast 3T3-L1 cell line. Our previous studies show that Les-2194 and Les-3640 increased reactive oxygen species (ROS) production in the SCC-15 cell line. Furthermore, both Les-2194 and Les-3640 decrease cell viability (measured by the resazurin reduction assay) and increase cell apoptosis (measured as caspase-3 activity) (Szychowski et al. 2017b). Moreover, our study shows that both Les-2194 and Les-3640 act in a PPARγ-dependent way (Szychowski et al. 2017a). Resveratrol, which is known as a PPARγ agonist, has been described to inhibit AhR-induced cytochrome CYP1A1 enzyme activity and expression (Ciolino and Yeh 1999). Based on this, the next stage of our study was to check the impact of 1 μM Les-2194, Les-3640, Les-5935, or Les-6166 on expression of *Cyp1a1*, *Cyp1b1*, *Cyp1a2*, and *Cyp2b10* mRNA in the 3T3-L1 cell line. In the experiments, 1 μM βNF, i.e., a known agonist of the AhR receptor, was used as a positive control. The results showed that βNF decreased the expression of *AhR* receptor mRNA but increased the expression of the AhR-dependent genes *Cyp1a1*, *Cyp1b1*, and *Cyp1a2*. Unfortunately, no expression of *Cyp2b10* was detected in the experiments. As shown by literature data, the cyp2b10 expression in the 3T3 cell line is very low and decreases during cell differentiation (Roth et al. 2008; Mesnier et al. 2015; Kong et al. 2018). Therefore, this may be the reason why we did not observe *Cyp2b10* mRNA expression in the 3T3-L1 cell line.

They demonstrated that Les-2194 affected the expression of *AhR*, *Cyp1a1*, *Cyp1b1*, and *Cyp1a2* mRNA in a similar way to βNF. However, Les-3640 increased only the *Cyp1a1* mRNA expression, while Les-5935 decreased the mRNA expression of this enzyme. The Les-6166 compound decreased only the expression of *AhR* receptor mRNA. It is important to underline that only βNF and Les-2194 induced the expression of *Cyp1a2* mRNA in the 3T3-L1 cell line.

Our previous study demonstrated that Les-2194 and Les-3640 act in a PPARγ-dependent way in SCC-15 cells and similar to known PPARγ agonists (rosiglitazone and pioglitazone) (Szychowski et al. 2017a). Moreover, it was described that PPARγ agonists could activate the expression of CYP1A1 in mouse and human cells *in vitro* (Kim et al. 2008; Wójtowicz et al. 2017), which is probably possible due to the crosstalk of the PPARγ and AhR receptor pathways (Wójtowicz et al. 2017). Unfortunately, the data on the role of PPARγ agonists in the expression of *CYP1A1*, *CYP1B1*, or *CYP2B1* mRNA (in mouse *Cyp2b10*) are very limited. However, since AhR control the expression of, e.g., *CYP1A1*, *CYP1A2*, and *CYP1B1* (Beischlag et al. 2008), it can be assumed that PPARγ agonists should affect the expression of these genes as well.

On the other hand, it has been reported that the PPARα agonist ciprofibrate can inhibit the expression of Cyp1a1 and Cyp1a2 mRNA and protein by interfering with AhR-dependent signaling in rat liver (Gallagher et al. 1995; Shaban et al. 2004). Such data suggest that Les-5935 and Les-6166 may be at least weak PPARα agonists. The final part of our study was to evaluate the effect of Les-2194, Les-3640, Les-5935, or Les-6166 on the activity of Cyp1a1, Cyp1b1, Cyp1a2, and Cyp2b10 in the 3T3-L1 cell line. It has been shown in various studies that EROD activities can be related to both CYP1A1 and CYP1A2 isoenzymes (Zamaratskaia and Zlabek 2009). However, some authors have suggested that the EROD assay measures CYP1B1 activity as well (Deeni et al. 2013). The MROD assay has been applied for measurement of the CYP1A2 activity (Conaway et al. 1996), whereas the PROD assay has been used to measure the CYP2B activity (Séïde et al. 2016). Our data showed an increase in the EROD activity after 24 and 48 h in the 3T3-L1 cells exposed to 1 μM Les-2194, Les-5935, or Les-6166; however, Les-3640 increased the EROD activity only after 48 h. In the case of Les-2194, the data on the *Cyp1a1*, *Cyp1a1*, and *Cyp1b1* mRNA expression and activity measured by the EROD assay are consistent. The increase in the EROD activity induced by Les-6166 may be explained by the increase in the *Cyp1a1* mRNA expression. Unfortunately, in the case Les-5935 and Les-3640, the increased EROD activity is not supported by the expression. This phenomenon may be a feedback effect where high enzyme activity inhibits its expression (Zanger and Schwab 2013).

Our data showed that only Les-2194 and Les-5935 increased the MROD activity, while βNF, Les-3640, and Les-6166 decreased the activity in the 3T3-L1 cells. To date, MROD activity has been correlated with CYP1A2 expression (Conaway et al. 1996). However, in our experiments, we observed increased expression of *Cyp1a2* mRNA only after the treatment of the cells with Les-2194. It cannot be excluded that Les-5935 affects only the activity of Cyp1a2 without significant induction of mRNA expression.

Our data showed that Les-2194 and Les-5935 contributed to an increase in the PROD activity, while the Les-6266 compound decreased it at both time intervals. Interestingly, the experiments showed no expression of *Cyp2b10* in 3T3-L1. A single *CYP2B* gene, i.e., *CYP2B6*, has been described in humans. In turn, mice have five *Cyp2b* genes: Cyp2b9, *Cyp2b10*, *Cyp2b13*, *Cyp2b19*, and *Cyp2b23* (Nelson et al. 2004). In mice, the *Cyp2b9*, *Cyp2b10*, and *Cyp2b13* forms are primarily expressed in the liver (Finger et al. 2011). The most important is *Cyp2b10*, and its expression is regulated by CAR (Honkakoski et al. 1998; Wang et al. 2003; Kretschmer and Baldwin 2005). Therefore, it cannot be excluded that the Les- compounds affect other than *Cyp2b10* mRNA expression.

Recently, it has been reported that different thiazolidinone derivatives exerted CYP inhibition activity in several isoforms (CYP1A2, CYP3A4, and CYP2C19) at 10 μM, but there was no CYP inhibition activity of CYP2C9 and CYP2D6 in human and rat liver microsomes (Ashraf et al. 2020). Unfortunately, the data on the CYP expression and/or activity after thiazolidinone treatment are limited.

It is well described that CYP1A1, CYP1A2, and CYP1B1 play an important role in the detoxication of environmental carcinogens and in the metabolic activation of dietary compounds with cancer preventive activity (Zanger and Schwab 2013; Alzahrani and Rajendran 2020). Moreover, the contribution of CYPs to cancer progression or prevention may depend on the balance of procarcinogen activation/detoxication and extrahepatic drug metabolism (Androutsopoulos et al. 2009). Similarly, CYP2B6 is involved in drug clearance (Zanger and Schwab 2013).

## Conclusions

Our study is the first to explore the role of the Les-2194, Les-3640, Les-5935, and Les-6166 compounds in the 3T3-L1 cell line. The experiments show that compounds Les-3640 and Les-6166 at the 50 and 100 μM concentrations activate caspase-3 in 3T3-L1 cells and can be toxic in these concentrations. Moreover, the study has shown that only Les-2194 acts similarly to our positive control βNF by affecting the mRNA expression of *AhR*, *Cyp1a1*, *Cyp1a2*, and *Cyp1b1* in the 3T3-L1 cell line. Interestingly, both Les-2194 and Les-5935 increased the activity of EROD, MROD, and PROD. Given the important role of the AhR receptor in the detoxification system and its crosstalk with the PPARγ receptor, which is crucial in adipocyte differentiation, more research in this field is needed to explain the mechanism of the Les-2194, Les-3640, Les-5935, or Les-6166 action on drug metabolism or the detoxification system and adipocyte differentiation.

## Supplementary Information

ESM 1 (DOC 143 kb)

ESM 2 (XLSX 61 kb)

## Abbreviations

AhR: Aryl hydrocarbon receptor
DMSO: Dimethyl sulfoxide
PBS: Phosphate-buffered saline
PPARs: Peroxisome proliferator-activated receptors
ROS: Reactive oxygen species
XO: Xanthine oxidize
αNF: Alpha-naphthoflavone
βNF: Beta-naphthoflavone
EROD: Ethoxyresorufin-O-deethylase
MROD: 7-Methoxyresorufin O-demethylation
PROD: Pentoxyresorufm O-dealkylase
DMEM: Dulbecco’s Modified Eagle’s Medium
