# Supplementary material for: Induction of Cyp450 enzymes by 4-thiazolidinone-based derivatives in 3T3-L1 cells in vitro
Source: Naunyn Schmiedebergs Arch Pharmacol. 2020 Nov 21;394(5):915–27. doi: 10.1007/s00210-020-02025-7 (PMC8102453; doi:10.1007/s00210-020-02025-7)
Supplement: Supplementary file 1 — (DOC 143 kb) [file 210_2020_2025_MOESM1_ESM.doc]

**Supplementary information**

Induction of Cyp450 enzymes by 4-thiazolidinone-based derivatives in 3T3 cells in vitro

Konrad A. Szychowski Ph.D. Sci.D.^1^, Bartosz Skóra MSc^1^, Anna Kryshchyshyn-Dylevych Ph.D.^3^, Danylo Kaminskyy Ph.D.^3^, Kamila Rybczyńska-Tkaczyk Ph.D.^2^, Roman Lesyk Ph.D., Sci.D., professor^1,3^, Jan Gmiński M.D, Ph.D., Sci.D., professor^1^

(family name is underlined)

^1^Department of Lifestyle Disorders and Regenerative Medicine, University of Information Technology and Management in Rzeszow, Sucharskiego 2, 35-225 Rzeszow, Poland

^2^Department of Environmental Microbiology, University of Life Sciences, Leszczyńskiego 7, 20-069 Lublin, Poland

^3^Department of Pharmaceutical, Organic and Bioorganic Chemistry, Danylo Halytsky Lviv National Medical University, Pekarska 69, Lviv 79010, Ukraine

**Corresponding author**

Konrad A. Szychowski

Department of Lifestyle Disorders and Regenerative Medicine, University of Information Technology and Management in Rzeszow, Sucharskiego 2, 35-225 Rzeszow, Poland, e-mail: konrad.szychowski@gmail.com

**Content:**

1. Spectral and analytical data for compounds **Les-5935** and **Les-6166** …………………… 2
2. *In vitro* anticancer assay of **Les-5935** ……………………………………………………. 3
3. Table S1. Influence of **Les-5935** on the Growth of Individual Tumor Cell Lines ……….. 4
4. References …………………………………………………………………………………. 6

**Spectral and analytical data for compounds Les-5935** and **Les-6166**

***General procedure for the synthesis of Les-5935***

Mixtures of 4-aminothiazol-2(5*H*)-one (5 mmol), 2-hydroxynaphthalene-1-carbaldehyde (5 mmol), and sodium acetate (5 mmol) were heated at reflux for 2 h in a mixture of AcOH and Ac_2_O (10 mL, 1:1). After cooling, the formed precipitate was filtered off, washed with AcOH, H_2_O, and EtOH, and re-crystallized from a mixture of DMF : EtOH (2:1)

***9H-Benzo[5,6]chromeno[2,3-d][1,3]thiazol-9-one* (Les-5935**). Yield 80%; mp >260°C. ^1^H NMR (400 MHz, DMSO-*d_6_*): δ 7.73 (t, 1H, *J* = 7.2 Hz, arom.), 7.83 (t, 1H, *J* = 7.2 Hz, arom.), 7.92 (d, 1H, *J* = 9.0 Hz, arom.), 8.15 (d, 1H, *J* = 7.8 Hz, arom.), 8.32 (d, 1H, *J* = 9.0 Hz, arom.), 8.64 (d, 1H, *J* = 8.3 Hz, arom.), 9.39 (s, 1H, arom.). ^13^C NMR (100 MHz, DMSO-*d_6_*): δ 116.1, 117.5, 123.1, 127.5, 128.0, 128.4, 129.0, 129.2, 129.4, 131.0, 134.0, 150.4, 175.2, 176.8. LCMS (ESI+) m/z 254.0 (M+H)^+^. Calcd. for C_14_H_7_NO_2_S: C, 66.39; H, 2.79; N, 5.53; Found: C, 66.20; H, 2.60; N, 5.70%.

***General procedure for the synthesis of Les-6166***

A mixture of 0.1 mole of 4-hydroxyphenylthiourea (0.1 mol), methyl 5-fluoro-3-formylindole-2-carboxylate (0.11 mol), chloroacetic acid (0.1 mol), and fused sodium acetate (0.1 mol) in 100 ml of acetic acid was heated under reflux for 3 h. The crystalline precipitate was filtered off, washed with acetic acid, water, ethanol, and diethyl ether, and then recrystallized from a mixture DMF-acetic acid (1:2).

***Methyl 5-fluoro-3-[2-(4-hydroxyanilino)-4-oxo-4,5-dihydro-1,3-thiazol-5-ylidenmethyl]-1H-2-indolecarboxylate*** (**Les-6166**). Yield 78%; mp 284-286°C. ^1^H NMR (400 MHz, DMSO-*d*_6_) δ 3.90, 3.93 (2*s, 3H, OCH_3_), 6.72, 6.82 (2*d, 2H, *J* = 8.6 Hz, arom.), 6.89 (d, 1H, *J* = 8.4 Hz, arom.), 7.14-7.36, 7.48-7.54 (2*m, 2H, arom.), 7.57 (d, 2H, *J* = 8.8 Hz, arom.), 8.11, 8.25 (2*s, 1H, =CH), 9.45, 9.56 (2*s, 1H, OH), 11.22, 12.04 (s, br.s, 1H, NH), 12.61, 12.65 (2*s, 1H, NH). ^13^C NMR (100 MHz, DMSO-*d_6_*): δ 52.8 (OCH_3_), 106.9, 116.1 (d, 6-C_ind_, *J*_CF_ = 24.9 Hz), 116.5 (d, 7-C_ind_, *J*_CF_ = 5.5 Hz), 122.5, 123.9, 124.4, 127.1, 127.6, 127.9, 129.9, 130.8, 155.2, 155.6, 156.1, 157.6 (d, 6-C, *J*_CF_ = 239.9 Hz), 161.2, 161.3, 169.5, 172.5, 180.4. LCMS (ESI+) m/z 412.0 (M+H)^+^. Calcd. for C_20_H_14_FN_3_O_4_S: C, 58.39; H, 3.43; N, 10.21; Found: C, 58.45; H, 3.35; N, 10.30%.

***In vitro* anticancer assay**

The primary anticancer assay was performed on a panel of approximately sixty human tumor cell lines derived from nine neoplastic diseases, in accordance with the protocol of the Drug Evaluation Branch, National Cancer Institute, Bethesda [1-4]. The cytotoxic and/or growth inhibitory effects of the most active selected compounds were tested *in vitro* against the full panel of human tumor cell lines at concentrations ranging from 10^-4^ to 10^-8^ M. A 48-h continuous drug exposure protocol was followed and an Sulforhodamine B (SRB) protein assay was used to estimate cell viability or growth.

The human tumor cell lines of the cancer screening panel are grown in RPMI 1640 medium containing 5% fetal bovine serum and 2 mM L-glutamine. For a typical screening experiment, cells are inoculated into 96 well microtiter plates in 100 μL at plating densities ranging from 5,000 to 40,000 cells/well depending on the doubling time of individual cell lines. After cell inoculation, the microtiter plates are incubated at 37° C, 5 % CO_2_, 95 % air and 100 % relative humidity for 24 h prior to addition of experimental drugs.

After 24 h, two plates of each cell line are fixed in situ with TCA, to represent a measurement of the cell population for each cell line at the time of drug addition (Tz). Experimental drugs are solubilized in dimethyl sulfoxide at 400-fold the desired final maximum test concentration and stored frozen prior to use. At the time of drug addition, an aliquot of frozen concentrate is thawed and diluted to twice the desired final maximum test concentration with complete medium containing 50 μg/mL gentamicin. Additional four, 10-fold or ½ log serial dilutions are made to provide a total of five drug concentrations plus control. Aliquots of 100 μL of these different drug dilutions are added to the appropriate microtiter wells already containing 100 μL of medium, resulting in the required final drug concentrations.

Following drug addition, the plates are incubated for an additional 48 h at 37°C, 5 % CO_2_, 95 % air, and 100 % relative humidity. For adherent cells, the assay is terminated by the addition of cold TCA. Cells are fixed in situ by the gentle addition of 50 μL of cold 50 % (w/v) TCA (final concentration, 10 % TCA) and incubated for 60 minutes at 4°C. The supernatant is discarded, and the plates are washed five times with tap water and air dried. SRB solution (100 μL) at 0.4 % (w/v) in 1 % acetic acid is added to each well, and plates are incubated for 10 minutes at room temperature. After staining, unbound dye is removed by washing five times with 1 % acetic acid and the plates are air dried. Bound stain is subsequently solubilized with 10 mM trizma base, and the absorbance is read on an automated plate reader at a wavelength of 515 nm. For suspension cells, the methodology is the same except that the assay is terminated by fixing settled cells at the bottom of the wells by gently adding 50 μL of 80 % TCA (final concentration, 16 % TCA). Using absorbance measurements [time zero (*Tz*), control growth in the absence of the drug (*C*), and test growth in the presence of the drug (*Ti*)], the percentage growth was calculated for each drug concentration. Percentage growth inhibition was calculated as:

[(*Ti* *- Tz*) */* (*C - T*z)] x 100 for concentrations for which *Ti* ≥ *Tz*,

[(*Ti - Tz) / Tz*] x 100 for concentrations for which *Ti* < *Tz*.

Dose response parameters (GI_50_, TGI) were calculated for each compound. Growth inhibition of 50% (GI_50_) was calculated from [(*Ti - Tz*)/(*C - Tz*)] x 100 = 50, which is the drug concentration resulting in a 50% lower net protein increase in the treated cells (measured by SRB staining) compared to the net protein increase seen in the control cells. The drug concentration resulting in total growth inhibition (TGI) was calculated from *Ti* = *Tz*. Values were calculated for each of these parameters if the level of activity was reached; however, if the effect was not reached or was excessive, the value for that parameter was expressed as higher or lower than the maximum or minimum concentration tested. The lowest values were obtained with the most sensitive cell lines. Compounds having GI_50_ values ≤100 μM were declared to be active.

Two independent experiments were performed. The selectivity indices for Les-5935 were calculated by dividing the full panel MG_MID (μM) by the value of the individual parameters for each cell line (μM). Ratios between 3 and 6 mean moderate selectivity, ratios greater than 6 indicate high selectivity toward the corresponding cell line, while compounds not meeting either of these criteria are rated nonselective [5].

**Table S1.** Influence of Les-5935 on the Growth of Individual Tumor Cell Lines. GI – growth inhibition, TGI – total growth inhibition, LC - lethal dosage.

| Disease | Cell line | GI_50_, μM | SI (GI_50_) | TGI, μM | SI (TGI) | LC_50_, μM | SI (LC_50_) |
| --- | --- | --- | --- | --- | --- | --- | --- |
| Leukemia | CCRF-CEM  HL-60(TB)  K-562  MOLT-4  RPMI-8226  SR | 0.844 / 1.16  0.572 / 3.50  0.425 / 0.650  0.808 / 4.06  84.1 / 30.8  - / 0.458 | 24.2 / 16.6  35.7 / 5.49  48.0 / 29.5  25.2 / 4.73  0.24 / 0.62  - / 41.9 | >100.0 / >100.0  >100.0 / >100.0  >100.0 / >100.0  >100.0 / >100.0  >100.0 / >100.0  - / >100.0 | - / -  - / -  - / -  - / -  - / -  - / - | >100.0 / >100.0  >100.0 / >100.0  >100.0 / >100.0  >100.0 / >100.0  >100.0 / >100.0  - / >100.0 | - / -  - / -  - / -  - / -  - / -  - / - |
|  | **MG_MID** | **17.3 / 6.77** | **1.18 / 2.84** | **>100.0** **/ >100.0** | **- / -** | **>100.0 / >100.0** | **- / -** |
| NSC lung cancer | A549/ATCC  EKVX  HOP-62  HOP-92  NCI-H266  NCI-H23  NCI-H322M  NCI-H460  NCI-H522 | 85.9 / >100.0  62.4 / 57.0  3.13 / 7.06  3.62 / 0.721  89.3 / 3.51  36.1 / 22.8  47.8 / 4.53  4.95 / 4.91  0.357 / 0.994 | 0.24 / -  0.33 / 0.34  6.52 / 2.72  5.64 / 26.6  0.23 / 5.47  0.57 / 0.84  0.43 / 4.24  4.12 / 3.91  57.1 / 19.3 | >100.0 / >100.0  >100.0 / >100.0  62.5 / >100.0  >100.0 / 33.4  >100.0 / >100.0  >100.0 / >100.0  >100.0 / >100.0  >100.0 / >100.0  >100.0 / >100.0 | - / -  - / -  1.46 / -  - / 2.72  - / -  - / -  - / -  - / -  - / - | >100.0 / >100.0  >100.0 / >100.0  >100.0 / >100.0  >100.0 / >100.0  >100.0 / >100.0  >100.0 / >100.0  >100.0 / >100.0  >100.0 / >100.0  >100.0 / >100.0 | - / -  - / -  - / -  - / -  - / -  - / -  - / -  - / -  - / - |
|  | **MG_MID** | **37.1 / 22.4** | **0.55 / 0.85** | **95.8 / 92.6** | **0.95 / 0.98** | **>100.0** **/ >100.0** | **- / -** |
| Colon cancer | COLO 205  HCC-2998  HCT-116  HCT-15  HT29  KM12  SW-620 | 16.3 / -  >100.0 / >100.0  0.659 / 1.97  20.9 / >100.0  61.6 / 5.28  0.946 / 0.976  0.634 / 1.75 | 1.25 / -  - / -  31.0 / 9.75  0.98 / -  0.33 / 3.64  21.6 / 19.7  32.2 / 11.0 | >100.0 / -  >100.0 / >100.0  >100.0 / >100.0  >100.0 / >100.0  >100.0 / >100.0  >100.0 / >100.0  >100.0 / >100.0 | - / -  - / -  - / -  - / -  - / -  - / -  - / - | >100.0 / >100.0  >100.0 / >100.0  >100.0 / >100.0  >100.0 / >100.0  >100.0 / >100.0  >100.0 / >100.0  >100.0 / >100.0 | - / -  - / -  - / -  - / -  - / -  - / -  - / - |
|  | **MG_MID** | **28.7 / 35.0** | **0.71 / 0.55** | **>100.0 / >100.0** | **- / -** | **>100.0** **/ >100.0** | **- / -** |
| CNS cancer | SF-268  SF-295  SF-539  SNB-19  SNB-75  U251 | 11.5 / 5.52  3.26 / 2.69  1.23 / 2.03  9.21 / 4.92  0.209 / 1.08  6.47 / 2.99 | 1.77 / 3.48  6.26 / 7.14  16.6 / 9.46  2.21 / 3.90  97.6 / 17.8  3.15 / 6.42 | >100.0 / >100.0  >100.0 / 85.9  >100.0 / >100.0  >100.0 / >100.0  7.13 / 14.4  >100.0 / >100.0 | - / -  - / 1.06  - / -  - / -  12.8 / 6.30  - / - | >100.0 / >100.0  >100.0 / >100.0  >100.0 / >100.0  >100.0 / >100.0  >100.0 / >100.0  >100.0 / >100.0 | - / -  - / -  - / -  - / -  - / -  - / - |
|  | **MG_MID** | **5.31 / 3.21** | **3.84 / 5.98** | **84.5 / 83.4** | **1.08 / 1.09** | **>100.0** **/ >100.0** | **- / -** |
| Melanoma | LOX IMVI  MALME-3M  M14  MDA-MB-435  SK-MEL-2  SK-MEL-28  SK-MEL-5  UACC-257  UACC-62 | 6.48 / 2.58  1.06 / 0.519  0.743 / 1.61  0.217 / 0.310  3.72 / 3.85  32.8 / 6.52  1.96 / 3.05  48.9 / >100.0  0.659 / 2.26 | 3.15 / 7.44  19.2 / 37.0  27.5 / 11.9  94.0 / 61.9  5.48 / 4.99  0.62 / 2.94  10.4 / 6.30  0.42 / -  31.0 / 8.50 | >100.0 / >100.0  >100.0 / >100.0  >100.0 / >100.0  0.561 / 1.09  >100.0 / >100.0  >100.0 / >100.0  >100.0 / >100.0  >100.0 / >100.0  >100.0 / >100.0 | - / -  - / -  - / -  1767.2 / 83.21  - / -  - / -  - / -  - / -  - / - | >100.0 / >100.0  >100.0 / >100.0  >100.0 / >100.0  61.6 / >100.0  >100.0 / >100.0  >100.0 / >100.0  >100.0 / >100.0  >100.0 / >100.0  >100.0 / >100.0 | - / -  - / -  - / -  1.61 / -  - / -  - / -  - / -  - / -  - / - |
|  | **MG_MID** | **10.7 / 13.4** | **1.91 / 1.43** | **89.5 / 89.0** | **1.02 / 1.02** | **95.7 / >100.0** | **1.03 / -** |
| Ovarian cancer | IGROV1  OVCAR-3  OVCAR-4  OVCAR-5  OVCAR-8  NCI/ADR-RES  SK-OV-3 | 17.6 / 5.88  0.429 / 0.488  58.4 / -  >100.0 / >100.0  30.9 / 28.0  >100.0 / >100.0  15.1 / - | 1.16 / 3.27  47.6 / 39.3  0.35 / -  - / -  0.66 / 0.69  - / -  1.35 / - | >100.0 / >100.0  >100.0 / 5.61  >100.0 / >100.0  >100.0 / >100.0  >100.0 / >100.0  >100.0 / >100.0  >100.0 / - | - / -  - / 16.2  - / -  - / -  - / -  - / -  - / - | >100.0 / >100.0  >100.0 / >100.0  >100.0 / >100.0  >100.0 / >100.0  >100.0 / >100.0  >100.0 / >100.0  >100.0 / - | - / -  - / -  - / -  - / -  - / -  - / -  - / - |
|  | **MG_MID** | **46.1 / 46.9** | **0.44 / 0.41** | **>100.0**  **/ 84.3** | **- / 1.08** | **>100 / >100.0** | **- / -** |
| Renal cancer | 786-0  A498  ACHN  CAKI-1  RXF 393  SN12C  TK-10  UO-31 | 1.27 / 8.34  0.184 / -  27.9 / 53.4  0.689 / 1.94  2.96 / 1.13  16.6 / 25.0  6.45 / >100.0  33.7 / - | 16.1 / 2.30  110.9 / -  0.73 / 0.34  29.2 / 9.90  6.89 / 17.0  1.23 / 0.77  3.16 / -  0.61 / - | >100.0 / >100.0  7.86 / -  >100.0 / >100.0  >100.0 / >100.0  52.2 / 8.96  >100.0 / >100.0  >100.0 / >100.0  >100.0 / >100.0 | - / -  11.6 / -  - / -  - / -  1.75 / 10.1  - / -  - / -  - / - | >100. 0/ >100.0  >100.0 / -  >100.0 / >100.0  >100.0 / >100.0  >100.0 / >100.0  >100.0 / >100.0  >100.0 / >100.0  >100.0 / >100.0 | - / 1.04  - / -  - / -  - / -  - / -  - / -  - / -  - / - |
|  | **MG_MID** | **11.2 / 31.6** | **1.82 / 0.61** | **82.5 / 87.0** | **1.11 / 1.04** | **>100.0** **/ >100.0** | **- / -** |
| Prostate Cancer | PC-3  DU-145 | 2.85 / 3.49  18.7 / 7.02 | 7.16 / 5.50  1.09 / 2.74 | >100.0 / >100.0  >100.0 / >100.0 | - / -  - / - | >100.0 / >100.0  >100.0 / >100.0 | - / -  - / - |
|  | **MG_MID** | **10.8 / 5.26** | **1.89 / 3.65** | **>100.0** **/ >100.0** | **- / -** | **>100 / >100.0** | **- / -** |
| Breast cancer | MCF7  MDA-MB-231/ATCC  HS 578T  BT-549  T-47D  MDA-MB-468 | 0.371 / 0.633  10.6 / 14.6  0.371 / 0.749  4.97 / 3.81  1.98 / 6.21  0.390 / 0.780 | 55.0 / 30.3  1.92 / 1.32  55.0 / 25.6  4.10 / 5.04  10.3 / 3.09  52.3 / 24.6 | >100.0 / >100.0  >100.0 / >100.0  38.2 / >100.0  >100.0 / >100.0  >100.0 / >100.0  18.7 / 23.0 | - / -  - / -  2.39 / -  - / -  - / -  4.89 / 3.94 | >100.0 / >100.0  >100.0 / >100.0  >100.0 / >100.0  >100.0 / >100.0  >100.0 / >100.0  >100.0 / >100.0 | - / -  - / -  - / -  - / -  - / -  -/- |
|  | **MG_MID** | **3.11 / 4.46** | **6.56 / 4.30** | **76.2 / 87.2** | **1.20 / 1.04** | **>100.0** **/ >100** | **- / -** |
| **MG_MID** |  | **20.4 / 19.2** |  | **91.4 / 90.7** |  | **99.3 / >100.0** |  |

**References**

1. A. Monks, D. Scudiero, P. Skehan, R. Shoemaker, K. Paull, D. Vistica, C. Hose, J. Langley, P. Cronise, A. Vaigro-Wolff, M. Gray-Goodrich, H. Campbell, J. Mayo, M. Boyd, Feasibility of a high-flux anticancer drug screen using a diverse panel of cultured human tumor cell lines, J. Nat. Cancer Inst. 83 (11) (1991) 757-766. https://doi.org/10.1093/jnci/83.11.757.
2. M.R. Boyd, K.D. Paull, Some practical considerations and applications of the national cancer institute in vitro anticancer drug discovery screen, Drug Dev. Res. 34 (1995) 91-109. https://doi.org/ 10.1002/ddr.430340203.
3. M.R. Boyd, In Cancer Drug Discovery and Development; Teicher, B. A. Ed.; Humana Press, 2 (1997) 23-43.
4. R.H. Shoemaker, The NCI60 human tumour cell line anticancer drug screen, Nature Reviews / Cancer 6 (2006) 813-823. https://doi.org/10.1038/nrc1951.
5. S. A. F. Rostom, Synthesis and in vitro antitumor evaluation of some indeno[1,2-c]pyrazol(in)es substituted with sulfonamide, sulfonylurea(-thiourea) pharmacophores, and some derived thiazole ring systems, Bioorg. Med. Chem. 14, 2006, 6475–6485. https://doi.org/10.1016/j.bmc.2006.06.020.
